# Autonomous Sensor for *In Situ* Measurements of Total Alkalinity in the Ocean

**DOI:** 10.1021/acssensors.4c02349

**Published:** 2025-02-12

**Authors:** Allison Schaap, Stathys Papadimitriou, Edward Mawji, John Walk, Emily Hammermeister, Matthew Mowlem, Socratis Loucaides

**Affiliations:** a National Oceanography Centre, European Way, Southampton SO15 3ZH, United Kingdom; b University of Southampton, NOC Campus, European Way, Southampton SO15 3ZH, United Kingdom; c Clearwater Sensors Ltd., Unit 208, Solent Business Centre, Millbrook Rd. W, Southampton SO15 0HW, United Kingdom

**Keywords:** total alkalinity, sensor, lab-on-chip, autonomous instruments, ocean carbonate system

## Abstract

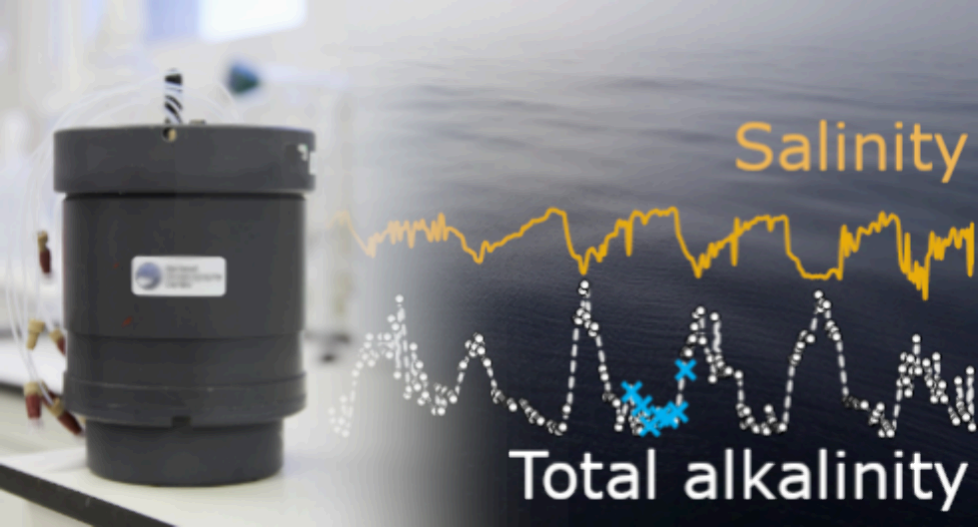

Total alkalinity (TA) is one of the measurable parameters that characterize the oceanic carbonate system. A high temporal and spatial frequency in TA data can lead to better measurements, modeling, and understanding of the carbon cycle in aquatic systems, providing insights into problems from global climate change to ecosystem functioning. However, there are very few autonomous technologies for *in situ* TA measurements, and none with field demonstrations below 2 m depth. To meet this need in marine observing capabilities, we present a submersible sensor for autonomous *in situ* TA measurements to full ocean depths. This sensor uses lab-on-a-chip technology to sample seawater and perform single-point open-cell titration with an optical measurement. It can carry multiple calibration materials on board, allowing for routine recalibration and quality checks in the field. The sensor was characterized in the laboratory and in a pressure testing facility to 600 bar (equivalent to 6 km depth) and deployed in a shallow estuary, on a lander at 120 m depth, and on an autonomous underwater vehicle. With a demonstrated precision and accuracy regularly better than 5 μmol kg^–1^ in field deployments, this sensor has the potential to dramatically expand our ability to perform long-term autonomous measurements of the marine carbonate system.

About half of anthropogenic CO_2_ emissions remain in the atmosphere contributing to the gradual warming of the planet, while the remaining half is taken up by the land and ocean at nearly equal proportions. About 10 Gt of CO_2_ is absorbed by the ocean every year,^1^ a fraction of which dissolves into carbonic acid (H_2_CO_3_) acidifying the ocean at a rate of 0.1 pH units per century.^2^ Ocean acidification threatens marine ecosystems by lowering the saturation states of carbonate minerals making seawater corrosive to the shells and skeletons of some marine organisms such as molluscs, corals, echinoderms, and calcifying planktons.^3,4^ Understanding the role of the ocean within the global carbon cycle and the progress and impact of ocean acidification requires widespread monitoring of the marine carbonate system on different temporal and spatial scales.^5^

The carbonate system can be characterized by constraining two of the five measurable parameters: total alkalinity (TA, defined as the equivalent concentration of proton acceptors over proton donors^6,7^), pH, total dissolved inorganic carbon (DIC), the fugacity of CO_2_ (*f*CO_2_), and the concentration of the carbonate ion (CO_3_^2–^).^8,9^ The propagated uncertainty of the calculated parameters is dependent on the choice of the input pair.^10^ Pairs that include TA (i.e., TA/DIC, TA/pH, and TA/*f*CO_2_) lead to smaller errors, and as a result, it is a top choice for characterizing the ocean carbon system.^11^

Although accurate measurements of seawater TA are routine in the laboratory, commercially available *in situ* sensors that meet ocean observing requirements (e.g., pressure tolerance, autonomy, and analytical performance) are lacking. Therefore, autonomous characterization of the carbonate system at sea is currently only possible using commercially available pH and *f*CO_2_ measurements, which lead to the largest propagated uncertainty in the calculated carbonate system parameters.^11^ Several published reviews of the commercial and research systems for the analysis of other marine carbonate parameters can be found elsewhere.^5,19,20^ In the absence of TA sensors, characterizations of the ocean carbonate system using autonomous platforms often rely on TA approximations using empirical stoichiometric relationships between TA and other parameters (i.e., salinity, temperature, nitrate, silicate, and apparent oxygen utilization).^12^ The validity of these approximations, however, varies regionally and can become less effective in coastal areas affected by freshwater inputs (i.e., under the influence of high-TA freshwater^12^) or areas with high rates of calcification such as coccolithophore blooms or coral reefs.^13^ Monitoring TA directly in these systems is important for characterizing ecosystem health and productivity or for monitoring anthropogenic impacts on the marine carbonate system. For example, measuring TA in coral reef systems at a high resolution is perhaps the most effective way in quantifying net community calcification rates, one of the prime indicators of reef health,^14^ but accurate measurements of calcification rates are hampered by the lack of technology that can accurately measure TA throughout diel cycles.^15^ This lack of sensors also presents challenges for evaluating ocean alkalinity enhancement (OAE), a strategy for atmospheric CO_2_ removal and ocean acidification mitigation. Autonomous TA sensors will be a key part of monitoring, reporting, and verification of OAE, establishing its effectiveness for carbon accounting and quantifying the efficiency required to scale up operations.^16,17^ The need is therefore high for autonomous instruments capable of performing highly accurate (0.1–0.5%) autonomous TA measurements *in situ* at a range of oceanic depths, with low enough size and power draw to allow integration on small autonomous vehicles, to overcome the spatiotemporal limitations of stationary or ship-based sampling.^18−20^

In laboratories, total alkalinity is generally determined by titrating a water sample against a known quantity of acid while monitoring the pH of the mixed solution. The standard operating procedure for TA determination in seawater^21^ is the multistep open-cell potentiometric titration using a strong acid titrant at constant temperature, with the excess CO_2_ generated from the titration of dissolved inorganic carbon removed from solution during the titration. Beyond the titration equivalence point, all proton acceptors present in seawater have been titrated, and bias from the presence of residual proton acceptors (i.e., bicarbonate ion) is negligible. The equivalence point and, from it, the sample TA can be determined from nonlinear fits to titration data within the end point pH range of 3.0 < pH < 3.5 given accurate knowledge of sample and added titrant masses, as well as titrant acidity.^21−24^ Titration pH is monitored by a glass electrode or a pH-sensitive indicator dye. Alternatively, a single-point titration can be used^22^ where a fixed, known quantity of the titrant is added to the sample, the CO_2_ is purged, and the solution pH is measured spectrophotometrically or potentiometrically. This technique, although simpler, requires *a priori* knowledge of the expected TA range to ensure that the quantity of the added titrant lowers the pH of the sample near pH ∼3.5. Since the range of TA values in open ocean water is generally fairly small (∼2100–2450 μmol kg^–1^^25^), this latter single-point approach is highly suitable for *in situ* technology and is the approach that we take in the sensor presented here. The desired precision and accuracy of oceanographic TA measurements are 2 or 10 μmol kg^–1^ for GOA-ON’s “climate” and “weather” goals, respectively,^26,27^ i.e., 0.1–0.5%, placing a stringing performance requirement on *in situ* instrumentation.

Few autonomous technologies to perform TA measurements *in situ* have been developed. The field-deployable SAMI-alk uses the spectrophotometric method with a monitored titration and is based on a design with a depth rating of 600 m.^28^ It can perform hourly measurements for a month, with each analysis consuming 4.5 mL of the titrant; its demonstration on a buoy at 1 m depth achieved an accuracy and precision of <1 and 15.7 μmol kg^–1^, respectively, compared to samples collected and analyzed in a lab.^28^ Two autonomous spectrophotometric TA sensors have been demonstrated in shallow submersible deployments. An automated microfluidic spectrophotometric TA analyzer (Dartmouth Ocean Technology, Inc.), using a design rated to 200 m depth, has been demonstrated in shallow (<2m depth) waters for 15 days in an estuary and 10 days at the mouth of a river with an accuracy of −0.17 ± 24 μmol kg^–1^ and a precision of 16 μmol kg^–1^.^29^ This microfluidic system consumed only 1 mL total of the sample and titrant per titration point. Another system, also using spectrophotometry, achieved much better precision (0.8 μmol kg^–1^). Shallow field deployments (1.2 m deep) showed an accuracy of 10.3 ± 2.8 μmol kg^–1^ with a single-point titration that used 32 mL of the sample and 0.6 mL of the titrant per measurement.^30^ An *in situ* instrument using an electrochemical approach to both sample acidification and end point pH detection has also been developed, achieving an initial precision of 0.5% (∼10–12 umol kg^–1^), which was improved to 2–10 μmol kg^–1^ with signal averaging.^31,32^ So far, this electrochemical technology has only been demonstrated in 1 m depth with an external seawater pump providing a fresh sample at the sensor surface, but it has the advantages of rapid measurements (∼1 Hz) using very little power. Despite these efforts, a need remains for deep sea-compatible autonomous technology, which can deliver TA measurements to the required analytical performance.

To address this need, we present a novel ruggedized submersible TA sensor based on a microfluidic lab-on-chip platform that can perform autonomous measurements *in situ* in the water. Its performance was assessed both in the laboratory and in pressure testing to the equivalent of 6 km depth, and in the field with a shallow estuarine deployment, on a lander at 120 m depth in the North Sea, and finally on an autonomous underwater vehicle to 590 m depth.

## Experimental Section

### Measurement Principle

**Figure 1:**
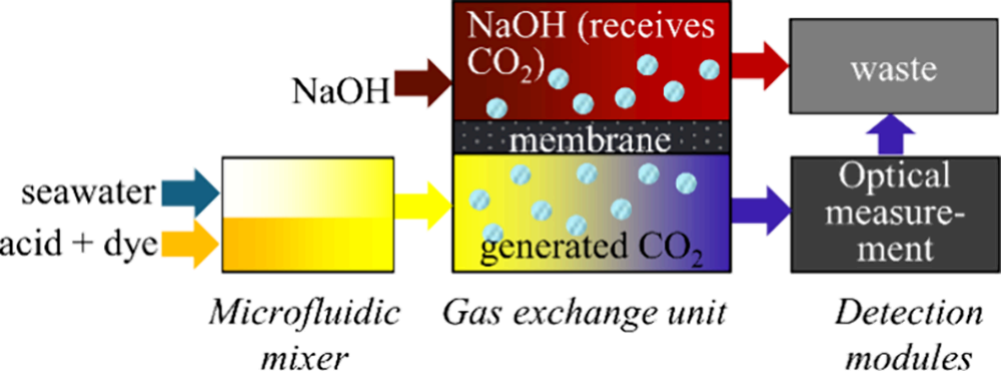
Overview of the single-point closed-cell method for measuring alkalinity.

The sensor uses the single-point open-cell method presented by Breland and Byrne^22^ and Li et al.^33^ Seawater is mixed with a salinity-matched titrant consisting of acid and a pH-sensitive indicator to bring the mixed sample to 3 < pH < 4 (Figure 1). The CO_2_ produced by this process is removed across a gas-permeable membrane into a recipient solution of NaOH. The resulting solution is sequentially illuminated in an optical cell by two LEDs with wavelengths (λ_1_ and λ_2_) at the absorption peaks of the pH indicator’s protonated and deprotonated forms.

A light detector at the end of the optical cell converts the amount of transmitted light to a voltage *V*, which yields measurements of optical absorption:
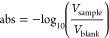
1where *V*_blank_ is the detector output while only sample water (without a titrant) is in the optical cell and *V*_sample_ is the detector output when the sample–titrant mixture is measured as described above.

The ratio of the absorbance values at the two wavelengths is normalized to temperature *T* = 25 °C using a linear temperature correction coefficient *c*_*T*_ to account for the temperature sensitivity of the pH indicator dye (Breland and Byrne):
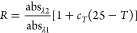
2

The solution pH at the titration end point is given by the equation
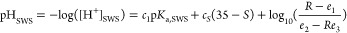
3which is derived from the equilibrium dissociation reaction of the protonated form of the dye expressed on the seawater proton scale (SWS), with p*K*_a,SWS_ = −log(*K*_a,SWS_), *K*_a,SWS_ = stoichiometric equilibrium second dissociation constant (mol/kg), *c*_*S*_ a linear salinity correction factor (Breland and Byrne), *c*_1_ an instrument and environmental-specific calibration factor, and *e*_1_, *e*_2_, and *e*_3_ are ratios of the molar attenuation coefficients of the dye determined at *T* = 25 °C and *S* = 35:
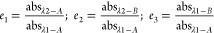
4

abs_λ*x–y*_ indicates the optical absorbance at wavelengths λ_1_ or λ_2_ of the dye in the deprotonated (B) or protonated (A) form.

The end point proton concentration (SWS) in the titrated sample is given in eq 5a. Equation 5b gives the end point excess proton concentration in the titrated sample in the presence of BPB, i.e., the sum of proton donors (acids) as defined for the zero proton condition for total alkalinity (Dickson^6^ and Wolf-Gladrow et al.^7^).
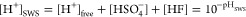
5a
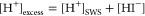
5b
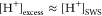
5cwith [H^+^]_free_ the free proton concentration, [HSO_4_^–^] the bisulfate ion concentration, [HF] the hydrogen fluoride concentration, and [HI^–^] the concentration of the protonated species of the dye. For the purpose of this study, HI^–^ is considered a minor component, such that the end point excess proton concentration in the titrated sample is given by eq 5c.

The sample TA (μmol/kg) is then computed as
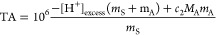
6where *m*_A_ is the titrant mass, *m*_s_ the sample mass, *M*_A_ the titrant acidity (in mol kg^–1^), and *c*_2_ a second instrument and environmental-specific calibration factor. Since the titrant, reference materials, and samples have matching salinity, we assume that any density changes (e.g., driven by ambient temperature) affect all three equally and use volume ratios defined by the pump geometry in place of masses. The processes for determining the calibration terms and titrant parameters are described in the “Calibration” section below.

### System Design and Hardware Implementation

**Figure 2:**
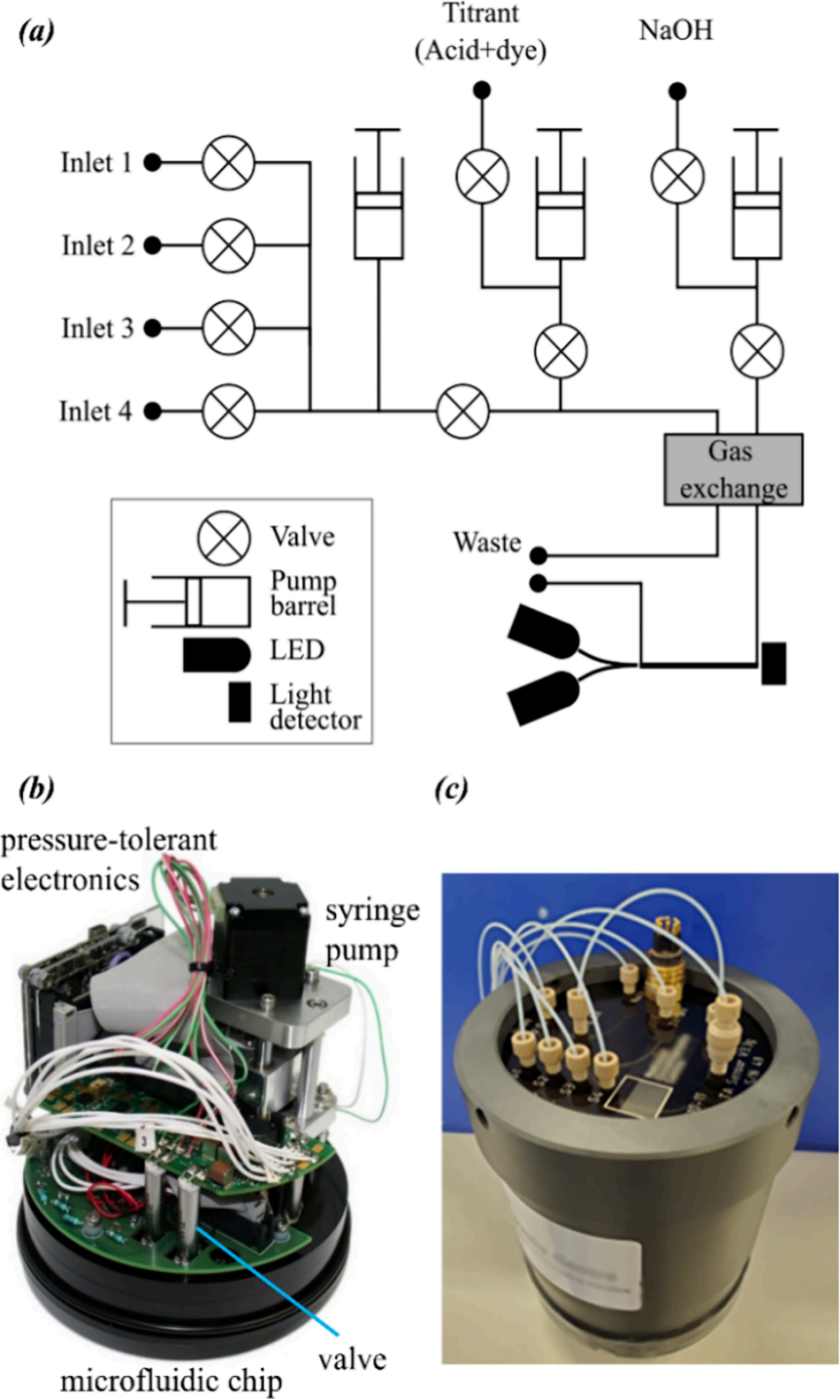
(a) Schematic of the sensor fluidic layout with inlets for samples labeled S1–S4 and the gas exchange unit GAS-X. (b) Photo of the interior of an assembled device showing the microfluidic chip with the valves, pump, and electronics. (c) Sensor in a waterproof housing; the orientation is upside down compared to image (b), and the visible outer face of the PMMA has tubing attached.

The TA sensor is built around a hardware platform,^34^ which has previously been implemented for other chemical assays including nitrate,^34,35^ phosphate,^34,36^ pH,^37^ and iron.^38^ The core of the device is a microfluidic chip consisting of three layers of tinted poly(methyl methacrylate) (PMMA), which are milled with microchannels, which serve as fluidic channels and optical cells (Figure 2a). The channels are 160 μm wide and 300 μm deep, except for the optical absorbance cells, which are 400 μm wide, 300 μm deep, and 15 mm long. After milling, the PMMA layers are solvent-bonded together,^39^ and valves, a syringe pump, and electronics are mounted directly onto the interior-facing side of the microfluidic chip (Figure 2b). The chip acts as an end-cap to a plastic waterproof pressure-compensating housing filled with mineral oil (Figure 2c). Reagents and reference materials are stored in flexible bags (Flexboy, Sartorius) outside the device. They and the sample inlet are connected to the microfluidic chip with tubing. The sample inlet tubing has a syringe filter attached to it to prevent particles from entering the instrument (Millipore Millex poly(ether sulfone) syringe filter, 0.45 μm pore size, 33 mm diameter). Once assembled, with a protective housing around the flexible bags, the entire instrument is 20 cm in diameter and 56 cm long and weighs 6 kg in air and <2 kg in water. For deployment in an underwater vehicle, it is possible to remove the protective housing around the flexible bags (relying on the vehicle faring for protection), which reduces the weight in water to 0.85 kg.

Fluids are pumped with a custom syringe pump (Figure 2b) comprising three barrels: a large barrel (inner diameter: 9.68 mm) fills with the sample and two smaller barrels (inner diameter: 3.26 mm) fill with the titrant and NaOH. The three plungers on the pump are all attached to a single metal plate, which is actuated by a stepper motor. This ensures that the volume ratio of the sample to titrant injected into the chip is an invariant 8.817, which is important for calculating TA (eq 6). During operation, the pumping rate of the sample is 960 μL/min, and that of the titrant and NaOH is 109 μL/min. The sensor has four inlet ports, each of which can be used for the intake of the sample or a reference material. Commercially available valves (LFNA1250125H, The Lee Company; Figure 2b) allow the fluid into the pump barrels and out into the chip and allow the user to set which material will be analyzed.

A tube-in-a-tube gas removal assembly based on the work of Li et al.^33^ is attached directly to the exterior of the microfluidic chip. An inner tube of gas-permeable Teflon AF2400 (inner diameter, 600 μm; outer diameter, 800 μm; BioGeneral, Inc.) contains 0.1 M NaOH, which readily absorbs CO_2_. This tube is inside a gas-impermeable PEEK tube (inner diameter, 1000 μm) through which the sample–titrant mixture is pumped. The CO_2_ produced during acidification is driven across the Teflon tube by the concentration gradient generated between the titrated sample and the NaOH solution. The volume of both the sample and NaOH in the gas removal assembly is ∼60 μL.

The optical absorbance measurements of the sample–titrant mixture take place in an optical cell (15 mm long, 400 μm wide, and 300 μm deep) with optical components glued into milled pockets on either end of the cell. The light from two LEDs, λ_1_ = 435 nm (LED430-06, Roithner Lasertechnik) and λ_2_ = 591 nm (C503B-AAN-CA0C0252-015; Cree, Inc.), is coupled into the cell by a Y-shaped microchannel (400 μm wide, 300 μm deep) filled with an optically transparent glue of higher refractive index than the surrounding PMMA, causing it to act as a light guide. A light-to-voltage converter (TSL257, AMS Technologies) at the end of the optical absorbance cell provides a readout of the light intensity.

The dissociation optical properties of the bromophenol blue (BPB) indicator dye used in the titration are temperature-sensitive, which necessitates a direct measurement of the temperature of the fluid inside the microchannels. Thermistors (527-P60BB203K, Amphenol Advanced Sensors) are positioned and sealed directly in the microchannel before and after the optical cell. Custom pressure-tolerant circuitry provides power management, communications, data handling and storage, and control of fluidic components. A constant-current control circuit powers the LEDs, and a 16-bit analog-to-digital converter reads the output of the light detectors and thermistors. The system is controlled by an SAM4L microprocessor (Microchip Technology). Exterior waterproof connectors (IE55 connectors, Teledyne Marine) enable the provision of external power and communications via USB or RS232 protocols. The USB connection allows for setup and control of the sensor with a custom graphical user interface. Following setup, the sensor can be directly started by the user, with data displayed in real time on a PC, or set to automatically start upon power up or at a specific time and date for field deployments. The system requires 10–18 V and consumes an average of 170 mA while operating, resulting in an average power consumption of 2 W with a 12 V power.

### Measurement Procedure

To perform a TA measurement, the sensor first measures an optical blank. The sample is withdrawn into the large barrel and injected into the chip (Figure 2a) to flush the device three times. After the third injection, the pump stops, and the background optical absorbance of the sample is measured at each wavelength (eq 1) as a blank. This process is then repeated but with the titrant and NaOH also injected. The NaOH is delivered to the recipient side of the gas exchange assembly. At the same time, the titrant mixes with the sample in an on-chip microfluidic mixer,^34^ and the mixed solution enters the donor side of the gas exchange assembly, where it remains for 60 s to degas.

Finally, the degassed solution is pumped into the optical absorbance cell for a pH measurement. One complete measurement cycle takes 10 min and uses 3.5 mL of the sample and 70 μL each of titrant and NaOH.

### Reagents and Reference Materials

The titrant was prepared gravimetrically from nonpurified BPB sodium salt (Sigma Aldrich, CAS 34725-61-6), HCl (37%, Honeywell Fluka), NaCl (SIGMA Aldrich), and the surfactant Tween-20 (SIGMA Aldrich, CAS 9005-64-5). All titrants were prepared with final concentrations of 0.36 mmol of BPB/L in the period 2018–2019 and 0.15 mmol of BPB/L thereafter, 37 g/L NaCl, 10 g/L Tween-20, and 0.021–0.030 mol/L HCl. The BPB concentration was reduced in later titrant batches to avoid dye recrystallization.

The titrant HCl concentration was selected for each deployment to suit the expected TA range. The analytical range of the system is controlled primarily by titrant acidity and the end point pH as indicated by eq 6. In terms of absolute concentration limits, for *M*_A_ = 0.026 mol/L, *S* = 35, and *T* = 25 °C, the range of TA that can be measured is from ∼1800 to 2540 μmol/kg. These represent the TA concentrations that have an end point pH (seawater scale) of between 3.5 and 3.0, respectively. Further information about this can be found in the Supporting Information.

BPB was selected as the pH indicator because its p*K*_a_ ≈ 3.7 at *T* = 25 °C and *S* = 35 PSU is within the pH range (pH 3–4) of the single end point pH of the titration method used on the sensor.^40^ Other dye options exist for measurements of the total alkalinity. Bromocrescol green (p*K*_a_ ≈ 4.4) and bromocrescol purple (p*K*_a_ ≈ 5.9) have both been used, for example,^22,23,28,33,41,42^ but these dyes have a p*K*_a_ value >4. By selecting a dye with p*K*_a_ close to the ideal pH of the measurement, we maximize instrument sensitivity to changes in sample TA by using the range where the indicator is most sensitive to pH changes.

Tween-20 was added to increase the solubility of the dye (which is a weak acid) in the acidic titrant solution. Experiments showed that from pH 3.8 to pH 1.5, the solubility of BPB decreased by ∼0.07 mmol/L per pH unit. The addition of the Tween increased the solubility of BPB in the titrant (pH 1.5) from ∼0.3 to >0.8 mmol/L at room temperature.

The NaOH solution was prepared from sodium hydroxide (CAS 1310-73-2) to a concentration of 0.1 M, far in excess of the concentration needed to fully remove the gaseous CO_2_ from the acidified sample solution.^43^

To test and calibrate the sensor, “CO_2_ in seawater” reference materials (hereafter referred to as “CRMs”) were purchased from the Marine Physical Laboratory, Scripps Institution of Oceanography, University of California San Diego, USA.^24^

### Calibrations

The temperature and salinity correction factors *c*_*T*_ and *c*_*S*_ (eqs 2 and 3) and the ratios *e*_1_, *e*_2_, and *e*_3_ of the BPB molar extinction coefficients (eqs 4) were determined in the laboratory and used to analyze field data. Two calibration variables, *c*_1_ and *c*_2_, were created (see eqs 3 and 6 and descriptions below) to allow for persistent recalibration in the field, even in the event of changes in the local environment (e.g., depth change), which may affect these parameters. Calibration processes to establish the values of each of these parameters were developed, tested, and implemented, with details in the Supporting Information.

### Sensor Testing, in the Lab and the Field

To assess the TA sensor’s reproducibility and accuracy, two CRMs, batch CRM172 (TA = 2217.4 μmol kg^–1^) and batch CRM162 (TA = 2403.72 μmol), were mixed in nine different gravimetrically determined mixing ratios. The TA of the CRMs and their aliquots was measured in five replicates each on a TA sensor, with parameters *c*_1_ and *c*_2_ calibrated against the certified TA of the two unmixed end-member CRMs.

The sensor was tested in the pressure testing facility at the UK’s National Oceanography Centre at 150, 300, 450, and 600 bar. The sensor and its consumables (reagents, two seawater solutions of known TA, and an uncharacterized seawater sample) were submersed in the testing chamber. The sensor was powered from outside the chamber via a bulkhead in the chamber lid. Three analyses of each solution were performed at each pressure.

The sensor’s performance was then validated in field trials in increasingly challenging environments.

For the first field deployment, the TA sensor was deployed for 10 days at a depth of 1 m in a tidal estuary in Southampton, UK. The sensor analyzed a sample every 15 min with two reference materials (CRM batches 172 and 162, Scripps Institution of Oceanography, USA) measured after every 9 samples to recalibrate values of *c*_1_ and *c*_2_. The sensor measurements were compared to the TA measured in 14 seawater samples; these were collected over 2 days using a Niskin bottle deployed on a rope directly next to the sensor. These samples were collected within 5 min of the sensor sample intake, stored in the dark in glass media bottles (DURAN) capped (gastight) with Teflon-lined screw-caps, and analyzed within a few hours to a few days.

A TA sensor was mounted onto a seabed lander deployed at 120 m depth from the RRS James Cook (expedition JC180, EU Horizon 2020 project STEMM-CCS) in the North Sea at 57° 59.7′N, 0° 0.37′W in May 2019.^44,45^ The sensor ran for 22 days off a battery pack. Two ships were in the vicinity (typically <1 km distance) during this deployment and took seawater samples using Niskin bottles on CTD. To provide further validation results, the instrument had an extra reference material (CRM batch 164, certified TA = 2309.3 μmol kg^–1^) on board. This material was not used for calibration but was reanalyzed regularly, interspersed throughout the seawater analysis, to quantify the sensor performance. Six samples were measured for every set of reference material measurements.

Lastly, a TA sensor was integrated onto the Autosub Long Range autonomous underwater vehicle (AUV)^46^ for a mission in the Celtic Sea on board RRS Discovery (expedition DY149). The AUV undertook transects of constant depth at 19, 95, 247, and 583 m along 47.5°N and longitude between 10.53 and 10.81°W for a total of 33 h. Seawater samples were collected using a CTD rosette and Niskin bottles from the vessel for comparison. Every CTD-collected sample was compared to the sensor measurement taken at the nearest longitude and depth for the analysis of the instrument performance. The reference materials were measured 15 times during this period, with nine samples measured between reference material reanalysis.

### Analysis of Seawater Samples Taken during Field Trials

To validate the sensor performance during field tests, the sensor measurements were compared to the TA measured in discrete seawater samples collected from the same location as the sensor. The samples were analyzed following standard operation procedures^21^ with a two-stage, open-cell potentiometric titration with ∼0.1 mol L^–1^ HCl at constant temperature (20 °C) using a Metrohm Ti-Touch 916 unit with an automatic buret, a pH meter, a Pt temperature probe, a Ag/AgCl/KCl reference electrode, and a glass indicator electrode calibrated with traceable buffers. Further details of the procedure are given in the Supporting Information.

## Results and Discussion

### Lab-Based Calibrations and Metrology Assessment

Six TA sensors were built and calibrated in the lab. Each sensor has two thermistors. Across the 12 calibrated thermistors, the root-mean-squared error (RMSE) of the fit ranged from 0.00488 to 0.101 °C, with a mean RMSE of 0.0268 °C. As an indication of the relevance of this error, when deployed in seawater at 7.8 °C and a TA of ∼2330 μmol kg^–1^, the largest RMSE would lead to a 1.0 μmol kg^–1^ TA error and the mean RMSE would lead to a TA error of 0.28 μmol kg^–1^.

The correction factors for temperature and salinity, *c*_*T*_ and *c*_*S*_, and the molar extinction coefficients varied across the sensors, indicating that the individual calibration of each system is required to achieve the needed performance. The means (±1σ) of the sensor-specific values across the 6 sensors were *c*_*T*_ = 0.00732 ± 0.00383/°C, *c*_S_ = 0.00461 ± 0.00151, *e*_1_ = 1.65 × 10^–3^ ± 5.94 × 10^–5^, *e*_2_ = 3.18 ± 0.217, and *e*_3_ = 4.60 × 10^–2^ ± 4.67 × 10^–3^ (*n* = 6 for all values).

**Figure 3:**
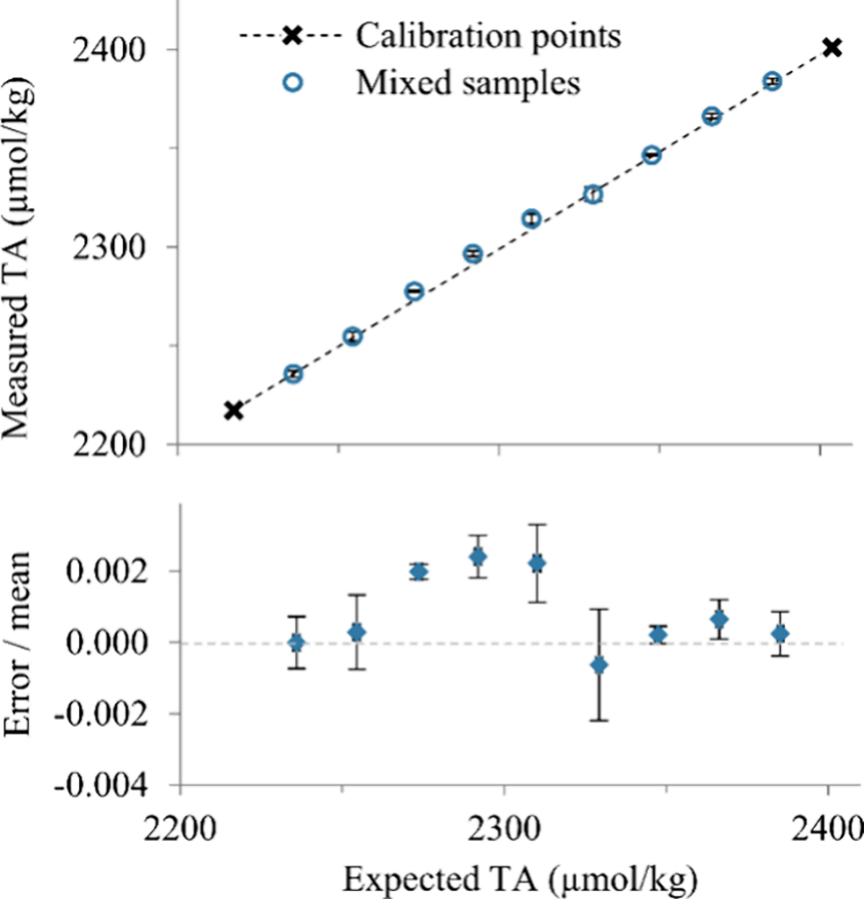
Total alkalinity from laboratory tests on two unmixed CRMs and their variably mixed aliquots. Top panel: calibration points indicate the end-member TA of the unmixed CRMs, which were used to set the *c*_1_ and *c*_2_ values. The blue circles represent the mean sensor-measured TA based on five replicate measurements of each mixed CRM aliquot. The straight dashed line represents the conservative TA mixing line. Bottom panel: the mean TA error (sensor-measured TA – expected TA from the mixing ratio) with error bars indicates the coefficient of variation.

The metrological performance of the instrument was validated against 5 repeat measurements of aliquots of reference materials. The standard deviation of all of the TA measurements from the mean of each sample was 1.68 μmol kg^–1^. The mean absolute error was 1.41 μmol kg^–1^ from the expected value (Figure 3).

### Pressure Testing

The sensor tested its pressure test to full ocean depth (600 bar), with no instrument issues arising at any pressure. The temperature inside the microfluidic channel remained between 21 and 23 °C throughout the test. The first measurement at each pressure was observed to be substantially different from the subsequent ones and was removed from analysis. After this, the sensor returned TA = 2246.1 ± 2.5 μmol kg^–1^ (*n* = 8 total) for the seawater sample across the pressure range. There was no relationship found between the TA measured and the pressure. However, we did observe a small pressure effect on the value of the coefficient *c*_1_, which appears to be driven by a pressure dependence of the optical properties of the pH indicator. This pressure dependence is effectively removed by our method of *in situ* recalibration.

### Shallow Estuarine Deployment (NOC Pontoon)

**Figure 4:**
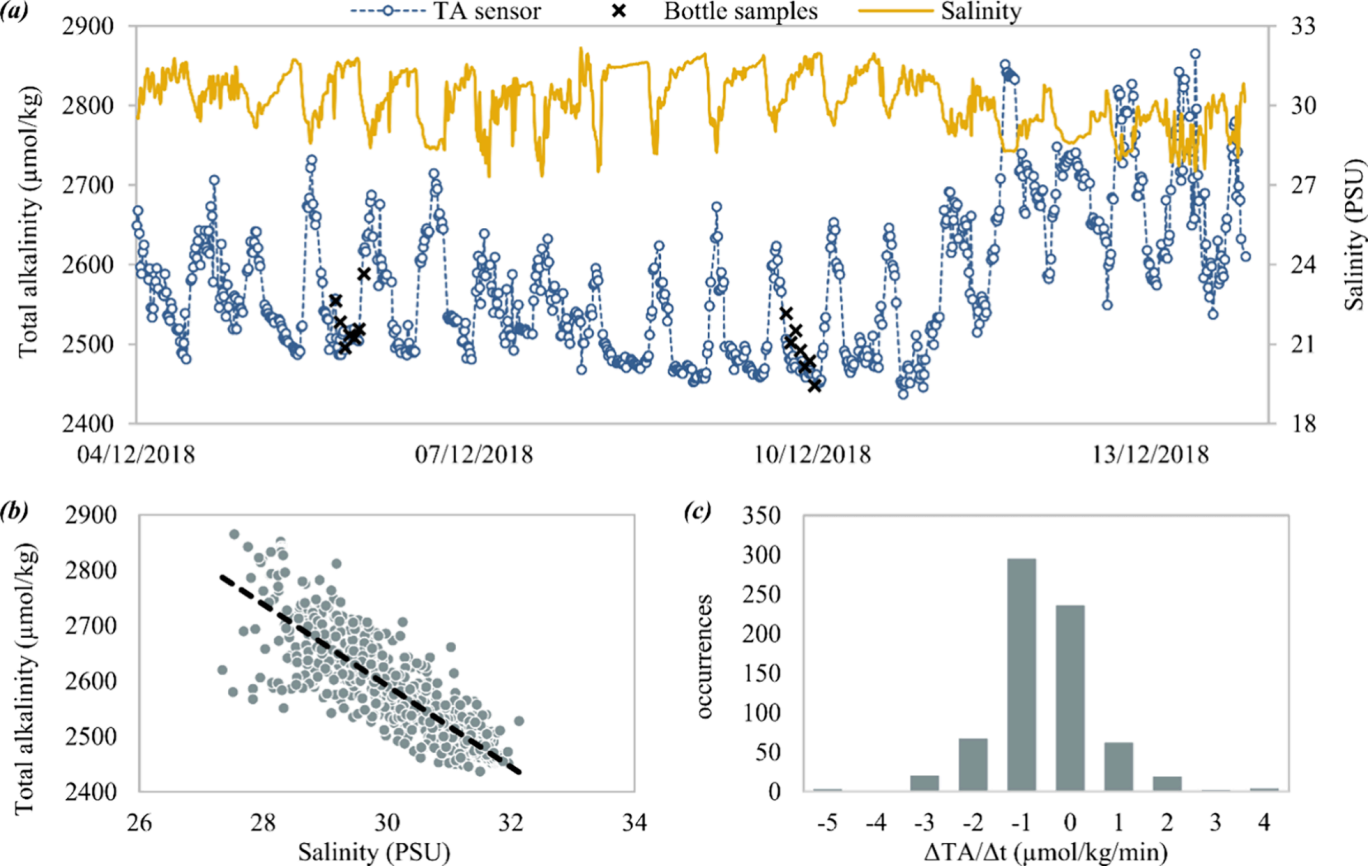
Results from a test deployment in a shallow estuary where high-TA freshwater from local rivers mixes with seawater. (a) Time series of the sensor TA (blue circles) and comparison with salinity and manually collected reference bottle samples. (b) Salinity–TA relationship, using the data from the TA sensor and a colocated CTD instrument (SBE37 Sea-Bird Scientific, Bellevue, USA). (c) Histogram of the observed rate of the change of TA with time, after a three-point moving average was applied to the sensor data to remove noise.

In the shallow estuarine deployment, the mean sensor-measured TA was 2577.0 μmol kg^–1^ with a range of 2437–2865 μmol kg^–1^ (Figure 4). The mean (±1σ) error (i.e., the difference between the nearest TA sensor and reference seawater TA measurement) was −5.8 ± 24.7 μmol kg^–1^ (*n* = 14). There was no statistically significant correlation between the errors and the salinity or temperature.

During the deployment, the water temperature ranged from 7.0 to 10.9°C. The salinity varied between 27.3 and 32.1, driven by the tidal mixing of fresh water from two local chalk rivers with seawater from the English Channel. The water in this estuary shows an inverse correlation between TA and salinity (linear regression fit: TA = 4796 – 73.5*S,r*^2^ = 0.73; Figure 4) due to the high alkalinity of the chalk rivers. The fitted relationship yields an estimate of TA ∼2220 μmol kg^–1^ for the seawater end-member (*S* = 35) and TA ∼4800 μmol kg^–1^ for the *S* = 0 freshwater end-member. This matches published measurements of the Itchen riverine water upstream of the tidal area (TA ∼ 4800–5000 μmol kg^–1^).^47^

This tidal system shows a high rate of change in TA. It is possible that some of the difference between the sensor values and the cosample data were driven by small offsets in timing between them. Based on a three-point moving average to the data to remove short-term and small-scale local mixing effects, the mean magnitude of the rate of change of TA was 1.13 μmol kg^–1^ min^–1^; during periods of rapid tidal flow, changes of more than ±5 μmol kg^–1^ min^–1^ were observed. This exemplifies the value of autonomous high-frequency sensing in dynamic environments and illustrates the necessity of accurately timing the collection of reference bottle samples for comparison to the sensor.

### Deployment in the North Sea on a Lander

During expedition JC180, over a 22-day period, the sensor made 152 measurements of the TA of the surrounding seawater, and 29 measurements each of CRM180 (TA = 2224 μmol kg^–1^) and CRM162 (TA = 2403 μmol kg^–1^) for calibration, and 29 measurements of an extra reference material (CRM batch 164, certified TA = 2309.3 μmol kg^–1^) interspersed throughout the seawater analysis, to quantify the performance of the sensor.

The sensor’s measurements (mean ± 1σ) of CRM batch 164 were 2311.4 ± 2.7 μmol kg^–1^ (*n* = 29), indicating that the instrument was accurate to ∼2 μmol kg^–1^. The measurements of the surrounding seawater showed a highly stable TA (mean ± 1σ) of 2333.6 ± 4.2 μmol kg^–1^ (*n* = 153). The water salinity was highly stable during this deployment, at (mean ± 1σ) 35.10 ± 0.023 (*n* = 153). Over the deployment, the TA of ship-based samples collected from depths >100 m ranged from 2301.1 to 2356.7 μmol kg^–1^ with a mean of 2319.8 μmol kg^–1^.

### AUV Deployment

**Figure 5:**
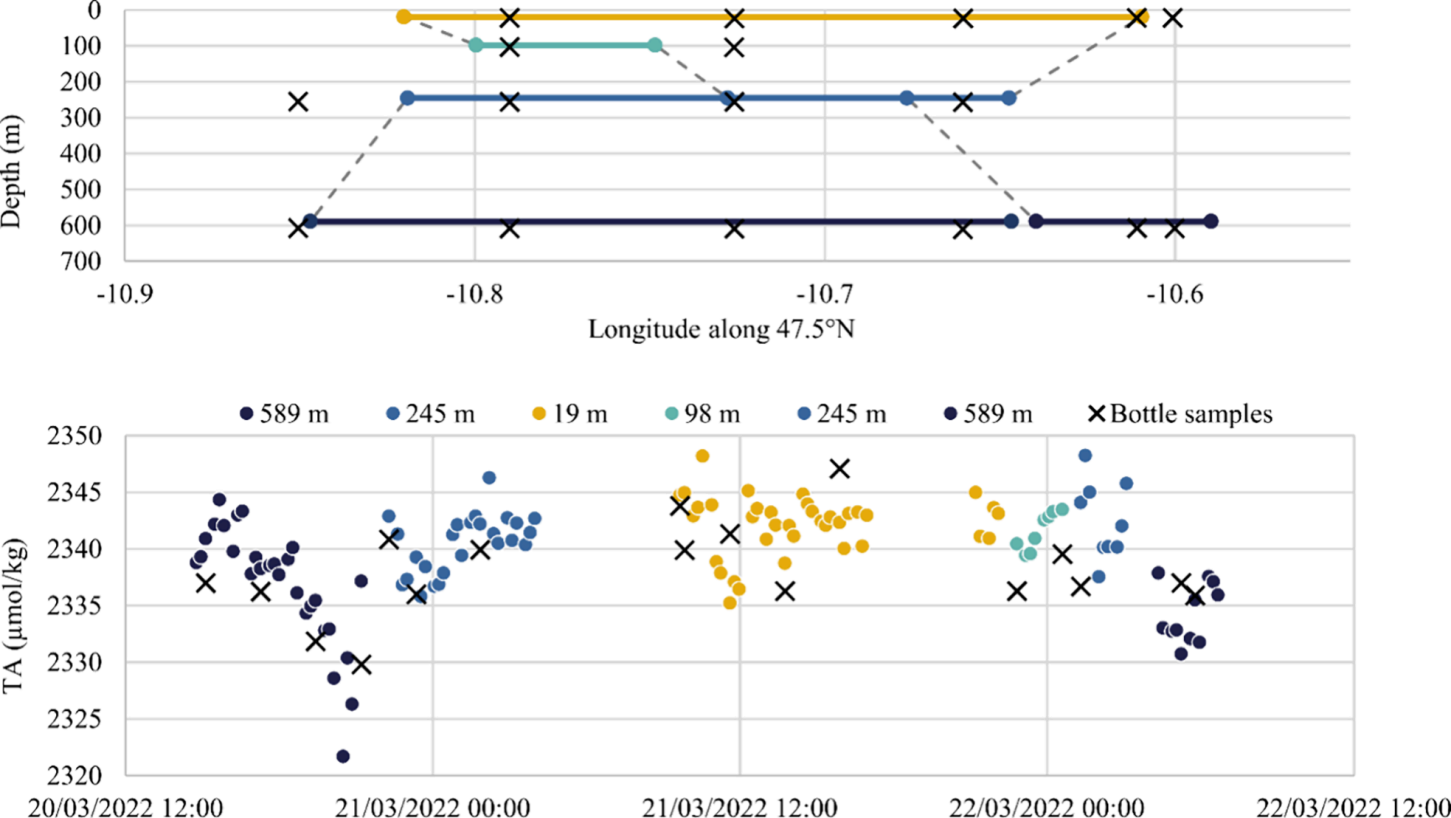
TA measurements taken with the sensor on an AUV and compared to measurements from colocated bottle samples taken from a ship. (Top plot) Longitude and depth of the vehicle (solid lines) and colocated bottle samples (black crosses). (Bottom plot) Time series of TA values measured by the sensor on the vehicle during constant-depth transits at the depths indicated, with TA values of colocated bottle samples for comparison.

With the sensor mounted on the AUV, data from three dives totaling 33 h yielded 144 TA measurements ranging from 2308.3 to 2349.9 μmol kg^–1^ (Figure 5). Seventeen ship-based CTD-collected samples were on average taken within 0.003 units of longitude, within 7% of depth, and within 1.2 days of the AUV-mounted sensor measurement. The difference between the bottle samples and the nearest matched sensor measurements (mean ± 1σ) was −1.8 ± 4.1 μmol kg^–1^ (*n* = 17).

There were no observable trends relating the difference between the sensor and bottle TA values and temperature, salinity, depth, or time. To explore the effect of depth on the calibration parameters of the sensor, the values of *c*_1_ and *c*_2_ were recalculated with every reanalysis of an on-board standard. This revealed a small correlation between *c*_1_ and depth (*c*_1_p*K*_a_ = 3.7369 – *d*(3 × 10^–5^) where *d* is depth in meters, *R*^2^ = 0.23). Overall, the *c*_1_p*K*_a_ and *c*_2_Ma values varied over a very small range, at (mean ± 1σ) 3.728 ± 0.016 and 0.0270 ± 0.0022 (*n* = 15), respectively.

### General Discussion

Each TA sensor is individually calibrated for optical parameters, temperature, and salinity. While a time-consuming procedure, the resulting calibrations thus take into account the effects of the changing environment on the entire system, including the dye, the individual components on the circuit boards, and the particular LEDs and photodetectors on the system. In the future, the characterization and use of purified dye or standard dye lots may allow a simplified calibration process, as has been established for a similar spectrophotometric pH sensor.^37^

The *in situ* recalibrations using reference materials allow the system to compensate for any other environmental sensitivities not yet fully characterized, e.g., depth. This approach also helps to compensate for simplifications made in the methodology, such as neglecting the contribution of [HI^*–*^] in the titrant to [H^+^]_excess_. Using a p*K*_a,SWS_ = 3.595 (mol/kg) based on p*K*_a,free_ = 3.697 (molality) in seawater at *S* = 35 and *T* = 25 °C (39), the effect of neglecting the contribution of [HI^*–*^] in the titrant to [H^+^]_excess_ (eq 5a) in the titrated sample can be calculated (eq 5c) to be equivalent to a pH_excess_ underestimate ranging from 0.005 to 0.026 log_10_ unit in the end point pH_SWS_ range of 3.0–3.5, leading to a TA underestimate ranging from 7 to 30 μmol/kg for the BPB concentration in the titrants used in this study and the *m*_S_/*m*_A_ of the sensors (see Reagents and Reference Materials). However, this effect applies to the calibration materials as well as to the seawater sample and is thus reduced by the calibration methodology.

While providing analytical value, this *in situ* recalibration process takes time and power and creates limitations for deployments in rapidly changing environmental conditions. It may be possible to reduce the frequency of in situ recalibrations based on observations of typical calibration drift under varying environmental conditions. This could occur on a preprogrammed schedule or be supplemented by adaptive recalibration triggered by a change in environmental conditions. For very long-term deployments, it may be necessary to consider alternative storage methods for the reagents and reference materials, as long-term storage tests of >1 year (data not shown here) do show some drift in the material over these time periods.

To date, biofouling has not yet been an issue on the sensor; the coastal test was done in December when biological growth was not strong and the other deployments were sufficiently deep that biofouling is a slow process. However, it is likely that during longer-term shallow deployments, consideration for antibiofouling measures would be necessary to prevent blocking of the intake filter.

The main observed causes of failures or errors of the sensors during testing and development were from the introduction of air bubbles, mechanical failures, or with lower analytical performance arising in settings with very rapidly changing temperature. Microfluidics are inherently sensitive to bubbles, and a bubble in the optical cell impairs the measurement. Fortunately, bubbles tend to be flushed out with the following pump injection; so, usually, only one data point is lost. However, care must be taken that the sensor inlet is not regularly exposed to air during a deployment, as the pump struggles to overcome the fluidic resistance caused by having air accumulate at the intake filters. Despite the temperature calibration, poorer data quality was seen in deployments when the ambient temperature changes extremely rapidly. For example, during the AUV dives, the ambient temperature changed rapidly upon entering the water, causing the internal sensor temperature to change by >1 °C between the optical measurements of the blank and mixed sample–titrant solution. This can be addressed by discarding data collected before the sensor has reached thermal equilibration with the ambient temperature, although future work to account for this may lead to suitable correction algorithms.

## Conclusions

Overall, the outcomes of the lab and field work demonstrate that this sensor regularly exceeds requirements for short-term “weather” TA uncertainties of <10 μmol kg^–1^ and is often at or close to the higher-specification “climate” uncertainties of <2 μmol kg^–1^. This has been demonstrated in a range of environments with a large range of salinities (20–36), temperatures (7–25 °C), and depths (0–6 km). Further studies of the causes and improvement of variability in the sensor are ongoing. However, this technology already offers a step change in capabilities for the *in situ* measurements of total alkalinity beyond that which is possible with other existing technologies in terms of observational frequency, sustained analytical performance, and autonomous deployment in inaccessible or remote oceanic areas.

## Supporting Information Available

Details of the procedures followed for lab analysis of bottle samples and of the calibration procedures created for the TA sensor (PDF)
