# Supplementary material for: Autonomous Sensor for In Situ Measurements of Total Alkalinity in the Ocean
Source: ACS Sens. 2025 Feb 12;10(2):795–803. doi: 10.1021/acssensors.4c02349 (PMC11877635; doi:10.1021/acssensors.4c02349)
Supplement: Supplementary file 1 — se4c02349_si_001.pdf [file se4c02349_si_001.pdf]

Supporting information for Publication:
An autonomous sensor for *in situ* measurements of total alkalinity in the ocean

Allison Schaap[a*], Stathys Papadimitriou[a], Edward Mawji[a], John Walk[a], Emily Hammermeister[a,b], Matthew Mowlem[a,c], Socratis Loucaides[a]

[a] National Oceanography Centre, European Way, Southampton, SO15 3ZH, United Kingdom

[b] University of Southampton, NOC campus, European Way, Southampton, SO15 3ZH, United Kingdom

[c] Clearwater Sensors Ltd., Unit 208, Solent Business Centre, Millbrook Rd W, Southampton SO15 0HW, United Kingdom

* Email: allison.schaap@noc.ac.uk

Analysis of discrete seawater samples taken during field trials

Discrete seawater samples were analyzed following standard operation procedures[21] with a two-stage, open-cell potentiometric titration with ~0.1 mol L^{-1} HCl at constant temperature (20 °C) using a Metrohm Ti-Touch 916 unit with automatic 5 mL burette, pH meter, Pt temperature probe, Ag/AgCl/KCl reference electrode, and glass indicator electrode calibrated with buffers traceable to SRM from NIST and PTB (pH 2.00, 4.01, 7.00, 8.99, and 10.01 at 25 °C; Certipur®, Merck).

A 150 mL jacketed titration vessel provided constant temperature control via a Thermo Scientific HAKE A10 chiller-recirculator with an AC150 heater. The TA was determined for pH = 3.5 – 3.0 from the Gran function

$$F_2 = (V_S + V_A)10^{-pH_{NIST}} = f_H(V_A - V_2)M_A \qquad [1]$$

where V_S = sample volume computed from mass-converted sample weight[21] and sample density computed from the equation of state of seawater[51] at the temperature of the titration, V_A = volume of added titrant (mL), f_H = the apparent activity coefficient of H^+, a function of solution composition and temperature, as well as the electrode used for the measurements[10], V_2 = volume of titrant added to reach the second equivalence point (mL), and M_A = titrant normality (mol/L). Based on the Gran function, linear regression of F_2 against V_A yields an intercept to slope ratio equivalent to V_2, and TA (µmol/L) = $10^6 M_A(V_2/V_S)$, with conversion to gravimetric units using sample density as above.[52] Titrations were conducted in replicate ~100 mL aliquots at constant pCO_2 (400 ppm CO_2) provided at a controlled rate (100 mL min^{-1}) via a CHELL CMD100 microprocessor controller and Hastings Mass Flow Control Valve, with a reproducibility better than 0.1%. A certified HCl solution of known density (batch A12, M_A = 0.0.099962 ± 0.000006 mol kg^{-1}, ρ_A = 1.02507 g/mL at 20°C, ~ 0.6 M NaCl; Marine Physical Laboratory, Scripps Institution of Oceanography) was used throughout these analyses. The in-house titrant batches were calibrated against the TA of an internal filtered seawater batch standardized on the A12 titrant. The uncertainty of the Gran titrations was 3 – 4 µmol kg^{-1}, estimated from the propagation in quadrature of the uncertainties of V_2 (from the linear fit to the titration data), M_A (from the M_A and density uncertainties on the certificate), V_S (from the weighing balance and seawater density uncertainties), and measurement reproducibility. Based on titrations of aliquots of CO_2 in Seawater Certified Reference Materials (CO_2 CRM batches 162, 164, and 172; Scripps Institution of Oceanography, University of California, San Diego, USA[24]), the TA offset (measured – certified value) was +6.1 ± 2.1 µmol kg^{-1} (n = 29), which was outside the measurement uncertainty and, thus, was treated as a systematic instrumentation bias for TA data correction.

TA sensor calibration procedures

Temperature calibration

Before assembly, thermistors and associated sensor electronics were individually calibrated between 2°C and 32°C in a highly temperature-stable water bath (Model 7009, Fluke) against a high accuracy temperature probe (F250, Automatic Systems Laboratories, Isotech). The temperature-voltage relationship was fitted with a cubic polynomial.

To obtain the temperature correction factor c_T (eq **Error! Reference source not found.**), the assembled sensor was submerged in the water bath and continuously measured T, *abs*1, and *abs*2 on a single solution while the temperature of the water bath was increased stepwise from 2°C to 26°C in 4°C intervals.

The temperature correction factor c_T is determined by linear regression as the absolute value of the slope of (*abs*2/*abs*1) vs {(*abs*2/*abs*1)(25 − T)} for a reference temperature of 25°C after linearization of Eq 2 by re-arrangement:

$$R = \frac{abs_2}{abs_1}(1 + c_T(25 - T) \Rightarrow \frac{abs_2}{abs_1} = R - c_T\frac{abs_2}{abs_1}(25 - T)$$

where *abs*2/*abs*1 = absorbance ratio measured by the sensor and T = optical cell temperature.

This correction factor encompasses the temperature sensitivity of the BPB dissociation equilibrium and the optical parameters (, i.e., pK_a in eq **Error! Reference source not found.**), and any temperature-dependent behaviour of the hardware (e.g. temperature-dependent output of the LEDs).

Salinity calibration

The salinity correction factor c_S was determined on each individual sensor system by repeated measurements of a dilution series of a sterilized filtered seawater standard (IAPSO standard seawater, Ocean Scientific International Ltd, UK) over the range 25<S<35. The TA of the undiluted standard was determined separately as outlined earlier for field samples, and the c1 and c2 values were taken from separate data collected elsewhere during the lab calibration process. The seawater was gravimetrically diluted with ultrapure water (Milli-Q®), creating a series of solutions of decreasing salinity (range: 25-35 PSU) and alkalinity (range: 1590-2225 µmol/kg)). After repeat TA measurements (n ≥ 4) of each solution using the sensor, the values of R (eq 2) and pH_{SWS} (eq 3) were determined. A linear fit of the difference between the expected TA (from the dilution factor) and the TA(S) of each solution from the measurement (eq 6) yields the correction factor c_S. While the sensitivity of TA to S is not linear with TA, a typical value of δTA/δS at TA=2300 µM/kg and 30<S<35 PSU is ~5 µmol/kg/PSU.

Titrant and optical calibration parameters

The values of c_1 and c_2 were calculated by solving eq 6 twice, using R values from two samples of different TA, their known TA values, and with c_1, c_2 as unknowns. These two known TA values were provided by the sensor analysing two certified reference materials (CRMs) during a deployment.

The values of e_1, e_2, and e_3 were determined by measuring the sensor optical response to a series of low pH and high pH saline synthetic solutions (e.g. with the indicator in its protonated or deprotonated form respectively) of different BPB concentrations, determined from gravimetric solution preparation. The BPB concentration ranged from 2.5 to 1025 μmol kg^{-1} (acidic solutions prepared with HCl to pH 1) and from 2.2 – 430 μmol kg^{-1} (basic solutions prepared with equimolal Tris-HCl buffer of pH = 8.25 at 20°C). All solutions were prepared gravimetrically. The BPB solutions were prepared from synthetic seawater stock solutions of practical salinity (S) 35 and from a 0.72 m (total ionic strength) stock solution of 10% (w/w) Tween-20, 0.62 m NaCl, and 0.004 m BPB. The synthetic seawater stock solutions were prepared as described in DelValls and Dickson (1998).[S1] The optical absorbance of a dye-blank aliquot of each stock solution were measured before and after each set of BPB solutions. These measurements were made in an environmental control chamber (DISCOVERY Angelantoni Test Technologies). The molar extinction coefficients – representing the absorbance per concentration unit of the protonated or deprotonated forms of the indicator - were determined by least-squares linear fit of the absorbance vs BPB concentration observations of each LED at each pH condition. The fitted slopes, which are in units of absorbance units per unit length per unit concentration, were used as the absorbance terms in eq **Error! Reference source not found.**:

$$e_1 = \frac{abs_{\lambda 2-A}}{abs_{\lambda 1-A}}; e_2 = \frac{abs_{\lambda 2-B}}{abs_{\lambda 1-A}}; e_3 = \frac{abs_{\lambda 1-B}}{abs_{\lambda 1-A}}$$

Preliminary investigation indicated that the dye absorptivity values were sensitive to temperature, so these measurements were done in a constant-temperature water bath to ensure the measurements were all at 25ºC, with temperature sensitivity covered by the system temperature calibration process.

(S1) DelValls, T. A.; Dickson, A. G. The pH of Buffers Based on 2-Amino-2-Hydroxymethyl-1,3-Propanediol ('Tris') in Synthetic Sea Water. *Deep Sea Res. Part I* **1998**, *45* (9), 1541–1554.

Sketch of optical absorbance cells and components

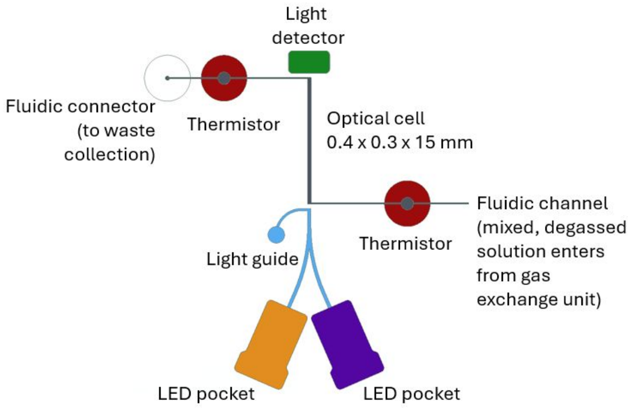

Figure S1. Sketch of a top-down (planar chip) view of the optical absorbance cell and surrounding components including LEDs, light guide, thermistors, and fluidic channels.

Selection of titrant acidity M_A for a deployment

The analytical range of the sensor is set by the acidity M_A of the titrant used for a deployment. To select the appropriate M_A, we use Equation 6 to calculate the acidity required to keep the endpoint pH between 3.0 and 3.5 for the expected range of TA values. This relationship is shown visually in Figure S2.

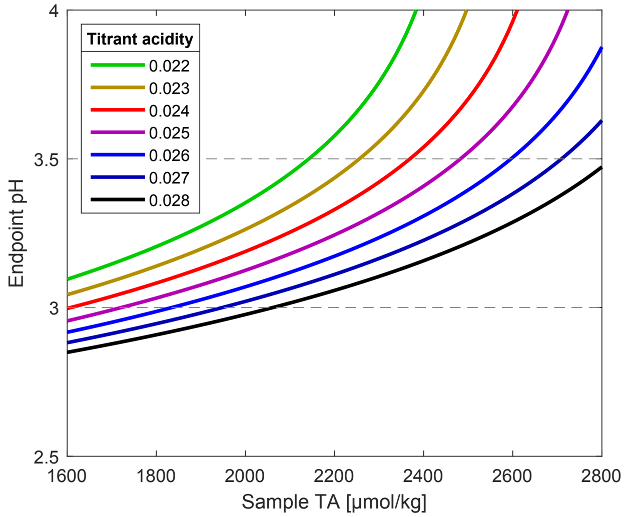

Figure S2. Relationship between sample TA, titrant acidity M_A, and end point pH of the mixed, degassed solution at the sample:titrant ratio used by the instrument. Each line on the plot represents one titrant acidity (in M). To prepare for a deployment, we identify the maximum and minimum expected TA concentrations, then identify a titrant concentration which results in the endpoint pH values remaining between 3.0 and 3.5.
